# miR-216b-5p promotes late apoptosis/necroptosis in trastuzumab-resistant SK-BR-3 cells

**DOI:** 10.55730/1300-0152.2655

**Published:** 2023-05-23

**Authors:** İştar Burcu DOLAPÇI, Senem NOYAN, Ayşegül YÜCEL POLAT, Hakan GÜRDAL, Bala GÜR DEDEOĞLU

**Affiliations:** 1 Biotechnology Institute, Ankara University, Ankara, Turkey; 2 Department of Medical Pharmacology, Faculty of Medicine, Ankara University, Ankara, Turkey

**Keywords:** miR-216b-5p, breast cancer, trastuzumab resistance

## Abstract

Breast cancer is the most common cancer in women. The human epidermal growth factor receptor 2 (HER2) overexpressing subtype is related to poor prognosis with an aggressive phenotype and is reported as one of the most commonly seen subtypes. Trastuzumab is prevalently used as a treatment method for HER2+ breast cancer however, resistance to the drug frequently occurs following the treatment. MicroRNAs (miRNAs) are 19–23 nucleotide long small RNAs, which regulate gene expression at post-transcriptional level and studies show that there are differentially expressed miRNAs between drug sensitive and resistant groups, indicating that they might have some key roles in drug effectiveness. In this study, the aim is to find out the role of miR-216b-5p in trastuzumab resistance.

SK-BR-3 cells developed resistance to trastuzumab after continuous treatment with increasing concentrations of the drug for 6 months. To investigate the effect of miR-216b-5p on cancer cell behavior in resistance state, proliferation, motility, and invasion capacities of these resistant cells were analyzed by xCELLigence real-time cell analyzer. To further understand the molecular mechanisms underlying the regulation of resistant SK-BR-3 cells by miR-216b-5p, microarray analysis was performed. Apoptosis analysis was also performed since the pathway enrichment analysis pointed out cell death related pathways.

The proliferation, motility, and invasion capacities of the miR-216b-5p transfected resistant cells were diminished compared to sensitive cells. We identified the necroptosis signaling pathway as the result of microarray and pathway enrichment analyses. *STAT1, IRF9, and PKR* were validated as the significant elements of the pathway, which are also the putative targets of miR-216b-5p. Our apoptosis analysis showed that a significant amount of trastuzumab resistant SK-BR-3 cells entered to late apoptosis/necrosis stage upon miR-216b-5p overexpression, it could be concluded that reexpression of miR-216b-5p sensitizes trastuzumab resistance through necroptosis in breast cancer.

## 1. Introduction

Breast cancer is the most common malignancy in women, having several subtypes. The morphology and grade of the tumor, tumor size, lymph node metastases status, and progesterone receptor (PR), estrogen receptor (ER), and human epidermal growth factor receptor 2 (HER2) expression profiles are considered together while evaluating the patients’ condition (Fragomeni, Sciallis, and Jeruss 2018; Goldhirsch et al., 2013). Breast cancer is mainly classified as luminal A, luminal B, HER2 expression increased (HER2+), and basal-like (BL) according to these gene expression levels (Perou et al., 2000; Sorlie et al., 2001). Increased HER2 expression is seen in approximately 25% of breast and ovarian cancers and indicates a poor prognosis (Slamon et al., 1989). HER2+ breast cancer is distinguished from other subtypes by showing increased resistance to certain hormonal treatments, a high recurrence rate, and an increased tendency towards brain metastasis (Gabos et al., 2006). Trastuzumab (Herceptin), a monoclonal antibody commonly used in the treatment of HER2+ breast cancers, is used to block the increase in HER2 signaling and the resulting activations of some related pathways, however, resistance to the drug following treatment for approximately one year is observed in the majority of HER2+ breast cancer patients (Pohlmann, Mayer, and Mernaugh 2009). Elucidation of the molecular mechanisms underlying the development of resistance is important in terms of developing more effective treatment methods against HER2+ breast cancer.

MicroRNAs (miRNAs) are small noncoding RNA molecules having functional roles in regulating gene expression through their ability to inhibit translation or cause mRNA degradation. It has been shown by many studies that miRNAs have key roles in cancer initiation, development, and metastasis. They affect tumor formation and development by regulating several cellular processes such as proliferation, differentiation, invasion, and motility (Di Leva and Croce 2010; Mendell and Olson 2012; Lin and Gregory 2015). Today, miRNA expression profiles playing roles in breast cancer formation and drug response continue to be defined, and this is important in terms of developing new and effective treatment methods that will eliminate the problem of trastuzumab resistance and paving the way for miRNA-based treatment options.

Previous studies performed in our lab (Cilek, Ozturk, and Gur Dedeoglu 2017) showed that miR-216b-5p has a key role in trastuzumab response. In this study, we first determined that miR-216b-5p has an anticancer effect on trastuzumab resistant SK-BR-3 cells, decreasing the proliferation, motility, and invasion rates of these cells and increasing the drug efficacy. The pathway enrichment analyses performed by using the differentially expressed gene lists obtained from our microarray data showed the role of necroptosis pathway in the anticancer effect of miR-216b-5p on resistant cells. We found that upregulation of miR-216b-5p in trastuzumab resistant SK-BR-3 cells results in the overexpression of *STAT1, IRF9*, and *PKR (EIF2AK2)*, which seems to promote necroptosis since the genes are at key points in necrosome formation. In addition, our experimental findings showing the apoptotic effect of miR-216b-5p on resistant SK-BR-3 cells supported our conclusion that miR-216b-5p exerts its anticancer effect on trastuzumab resistant SK-BR-3 cells through cell-death.

## 2. Materials and methods

### 2.1. Cell lines and culture

HER2+ breast cancer cell line SK-BR-3 (WT) was obtained from the American Type Culture Collection and the cells were maintained in McCoy’s 5A medium (Hyclone, USA) containing 10% FBS (Hyclone, USA), 1% penicillin/streptomycin (Lonza, Switzerland), and 1% L-glutamine (Lonza, Switzerland). SK-BR-3 cells were cultured in 5% CO_2_ at 37 °C. Trastuzumab resistant SK-BR-3 (TR) cells were additionally supplemented with 10μg/mL trastuzumab (Herceptin), which was purchased from Roche (Basel, Switzerland).

### 2.2. Resistant cell generation

The SK-BR-3 cell line resistant to trastuzumab (TR) was generated by exposing the cells to increasing concentrations of trastuzumab. The SK-BR-3 cell line was subjected to six weeks of treatment for each concentration of trastuzumab with rest periods until reaching an 80% of cell confluence. The treatment was started with a concentration of 0.5 μg/mL, and it was increased in each block of six weeks, until reaching a final concentration of 10 μg/mL. Stock solution of 150 mg trastuzumab (Roche) and further dilutions were prepared in PBS. The effect of trastuzumab on viability of WT and TR cells were evaluated at different concentrations (0.5 μg/mL, 2 μg/mL, 6 μg/mL, 10 μg/mL) using iCELLigence (ACEA Biosciences, USA), and final clones selected with 10mg/mL was used for further experiments.

### 2.3. Western blot

To confirm drug resistance state, cells were seeded and then exposed to different concentrations of trastuzumab. After double wash with cold PBS, the monolayers were scraped into lysis buffer (Complete Lysis-M kit, Roche) and protein isolation was completed according to the instructions manual. The protein concentration was determined using Coomasie Plus Bradford Assay (ThermoScientific) and used to estimate the volume of protein yielding 15 μg, which were boiled in Laemmli buffer and resolved on an 8% SDS-PAGE and transferred onto a PVDF membrane (L-08008-001, Advansta). Primary antibodies were: anti-ErbB2/HER2 (1:1000, ab8054, Abcam) and anti-Beta-actin (1:1000, 634801, Biolegend) was served as a loading control. Mouse secondary antibody (1:10,000, 7076S; Cell Signaling) conjugated with HRP (horseradish peroxidase) was added and kept at room temperature for 60 min. The immunoblot was developed with the WesternBright Sirius Kit (K-12043-D20, Advansta).

### 2.4. Transfection of the cells with miR-216b-5p mimic

WT and TR cells were seeded at a density of 25 × 10^4^ cells/well in six-well plates and transfection was performed 24 h later with Hi-perfect Transfection Reagent (Qiagen, 301705), and Allstar scrambled mimic negative control (Qiagen, SI03650318) or miR-216b-5p mimic (Qiagen, MSY0004959) for each well. Cells were harvested using a cell scraper in ice-cold PBS 48 h after transfection for RNA isolation.

### 2.5. RNA isolation and qRT-PCR

For miRNA expression analysis QIAzol reagent (Qiagen) was used for the isolation of total RNA according to manufacturer’s instructions and reverse transcribed to cDNA by using miScript II RT Kit (Qiagen). This cDNA was used to measure the miRNA expression to determine the transfection efficiency via qRT-PCR by using miScript SYBR Green PCR Kit (Qiagen). For the validation of mRNA targets total RNA isolated was reverse transcribed to cDNA by using Transcriptor High Fidelity cDNA Synthesis Kit (Roche) and validation was performed. The qRT-PCR reactions were performed with Roche Light Cycler 480 instrument and expression profiles were evaluated using the 2^−ΔΔCT^ method. U6 snRNA (MS00033740) and GAPDH were used as internal controls for miRNA and mRNA respectively. The sequence information of the miRNA and genes are given as Supplementary Table 1.

### 2.6. Proliferation, migration, and invasion assay

The xCELLigence RTCA system (ACEA Biosciences, Roche, Germany) was used to understand the effect of miR-216b-5p on the proliferation rate of WT and TR in real time. Cells were seeded at 2 × 10^4^ cells/well containing 10 ug/mL trastuzumab in E-plate 16 (ACEA Biosciences). The cells were monitored every 30 min.

The migration and invasion assays were performed as previously described (Noyan et al., 2021). Briefly, cell invasion analysis was performed with xCELLigence real-time cell analyzer. The miR-216b-5p mimic transfected cells were seeded with serum-free medium into the upper chamber of the ACEA Biosciences Inc. CIM-plate wells (Cat. no: 2801038), which was covered with a microporous membrane of matrigel separating the upper and lower chamber. The lower chamber was filled with chemoattractant that is culture medium supplemented with 10% FBS. Negative control (NC) wells contained serum free medium as the control of the experiment. Both cell invasion and motility were monitored for 24 h with xCELLigence realtime cell analyzer, using CIM-plate and measuring impedance-based signals. The same experimental set up was used without matrigel for motility experiments.

### 2.7. mRNA microarray and analysis

Total RNA isolated from scrambled control and miR-216b-5p mimic transfected WT and TR cells were used for the microarray experiment. GeneChip™ 3′ IVT PLUS Reagent Kit (Applied Biosystems, 902415) was used and the appropriate protocol for the Affymetrix U133 Plus 2.0 arrays was followed. The raw data was obtained in CEL files and analyzed by BRB-ArrayTools (Zhao and Simon 2008). The data was normalized by RMA (Robust Multiarray Average) normalization method (Irizarry et al., 2003) and differentially expressed mRNAs between miR-216b-5p transfected TR and WT compared to scrambled control were determined (fc ≥ 2) (p < 0.05).

The differentially expressed mRNAs (fc ≥ 2) (p < 0.05) in TR and in WT cells after miR-216b-5p transfection were compared by VENNY (Oliveros 2007) and only the differentially expressed mRNAs in trastuzumab resistant cells were used in pathway analysis. To perform the pathway enrichment of the differentially expressed genes, WebGestalt (WEB-based GEne SeT AnaLysis Toolkit) (B. Zhang, Kirov, and Snoddy, 2005; J. Wang et al., 2013) and KEGG (Kyoto Encyclopedia of Genes and Genomes) databases were used (Kanehisa and Goto 2000).

### 2.8. Apoptosis assay

Apoptosis assay was performed with NovoCyte^®^ Flow Cytometer (ACEA Biosciences, USA). WT and TR cells were seeded in 6-well plates at a density of 25 × 10^4^ cells/well and after 24 h the cells were transfected with miR-216b-5p mimic and scrambled control. Each experiment was performed as two biological replicates. The cells were harvested and washed with PBS 48 h posttransfection and were suspended in 1X Annexin V binding buffer (10 mM Hepes/NaOH; pH: 7.4; 140 mM NaCl, 2.5 mM CaCl_2_) in a concentration of 1 × 10^6^ cells/mL. Five μL Annexin V-FITC (640906, BioLegend) and five μL PI (421301, BioLegend) (0.5 μg/μL) were added and the cells were incubated in dark for 15 min. After the incubation period, 400 μL Annexin V binding buffer was added on the cells and data was obtained in an 1 h by NovoCyte^®^ Flow Cytometer (ACEA Biosciences, USA).

### 2.9. TCGA data analysis

The expression levels of miR-216-5p in the clinical samples were assessed in breast tumor tissues and normal samples using the TCGA Breast Cancer BRCA data set through the Xena Browser (Goldman et al., 2020). The BRCA data from TCGA was downloaded and individual data graphs were plotted for tumor/normal comparisons by using statistical analysis tool Minitab Software (Minitab LLC, State College, Pennsylvania, USA). The data was reanalyzed statistically and significance of the differences between the groups were indicated as p values.

### 2.10. Statistical analysis

To test the significance of the differences, student’s t-test was performed and p-value of less than 0.05 was considered as statistically significant. Two biological and two technical replicates were designed for each experiment.

## 3. Results

### 3.1. miR-216b-5p expression is lower in breast tumor tissues and in trastuzumab resistant HER2+ breast cancer cells

In our previous study we built a homogenous network model that focuses on the relationships between trastuzumab responsive miRNAs to explore the molecular mechanisms of trastuzumab treatment by extracting the miRNA-regulatory networks in breast cancer cell lines. In this study miR-216b-5p was found to be one of the hub proteins in trastuzumab response in SK-BR-3 cells (Cilek et al., 2017). Its central role in trastuzumab response and predicted targets of it led us to investigate the role of miR-216b-5p in trastuzumab resistance in SK-BR-3 cells.

**Figure 1:**
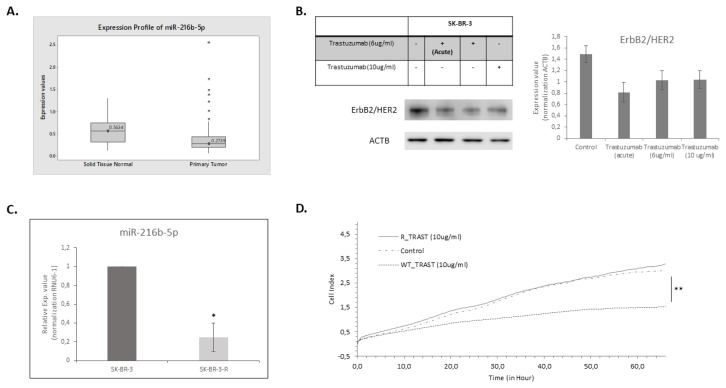
Lower miR-216b-5p expression is associated with tumor samples and trastuzumab resistance. A. Decreased level of miR-216b-5p was indicated in breast tumors compared to normal samples according to TCGA data analysis (Welch’s t-test; p = 0.01086, t = −2.743). B. ErbB2/HER2 protein expression level was decreased gradually when the SK-BR-3 cells gained resistance to trastuzumab. C. Real-time RT-PCR results showed that miR-216b-5p expression decreased as the cells gain resistance to trastuzumab. t-test; *p < 0.01, **p < 0.001 D. Cell viability experiments validated the resistance phenotype of SK-BR-3 cells.

First, to determine the clinical importance of miR-216b-5p, its expression level in breast tumors was investigated in TCGA Breast Cancer (BRCA) (n = 1240; tumor = 1101, normal = 139) dataset by using UCSC XenaBrowser. When the expression levels of the miRNA in tumor tissues and in normal tissues were compared, it was found that miR-216b-5p expression is significantly lower in tumor tissues (p = 0.01086) (Figure 1A).

To find out the expression pattern of miR-216b-5p upon trastuzumab resistance we developed trastuzumab resistant SK-BR-3 cells. The cells were continuously exposed to 10 μg/mL trastuzumab for one year until the cells acquired resistance. Throughout this process the HER2 protein level decreased in the resistant cells compared to sensitive ones (Figure 1B). The expression level analysis performed with these cells demonstrated that miR-216b-5p expression was lower in TR cells compared to WT cells (Figure 1C). Their resistance to trastuzumab was verified by proliferation assay via real time imaging of the cells and while the proliferation rate of the WT cells was significantly reduced after 10 mg/mL trastuzumab treatment compared to control cells (2 folds, p < 0.05), that of the resistant cells were not affected under these conditions (Figure 1D).

TCGA data together with qRT-PCR expression analysis showed its downregulation in tumor cells and this downregulation was even more when the cells acquired resistance to trastuzumab. These findings supported the potential role of miR-216b-5p in trastuzumab resistance and led us to further investigate its potential regulative role in resistance state.

### 3.2. miR-216b-5p overexpression decreases proliferation and motility of TR but not WT cells

**Figure 2:**
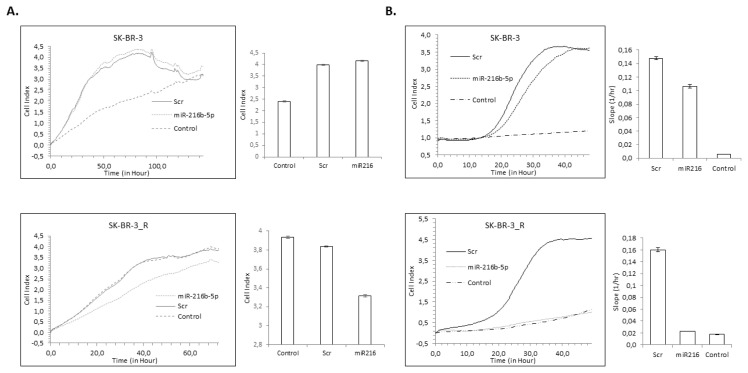
Up-regulation of miR-216b-5p caused a significant reduction in trastuzumab resistant SK-BR-3 cell viability and motility. SK-BR-3 (WT) and SK-BR-3-R (TR) cells were transfected with 25nM of miR-216b-5p mimic or scrambled control (scr), then exposed to 10 mg/mL of trastuzumab and their viability was assessed using iCELLigence E-plate (A). The percentage of motile cells was calculated with respect to negative control cells that are FBS free (*p < 0.05) (B).

When both WT and TR cells were transfected with miR-216b-5p mimic the proliferation rate of mimic transfected WT cells were not affected compared to scramble control transfected SK-BR-3 cells (Figure 2A). On the other hand, miR-216b-5p mimic transfection significantly diminished the proliferation rate of TR cells compared to control cells. Interestingly the resistant cells became sensitive to trastuzumab upon miR-216b-5p overexpression via mimic transfection (Figure 2A).

We observed similar results in motility experiments and overexpression of miR-216b-5p significantly decreased the motility and the invasion capacity (Figure 2B and Supplementary S1) of the TR cells but the motility of WT cells was not affected from miR-216b-5p transfection (Figure 2B).

### 3.3. miR-216b-5p promotes late apoptosis/necroptosis in trastuzumab resistant cells by regulating the expression of STAT1, IRF9, and PKR

**Figure 3:**
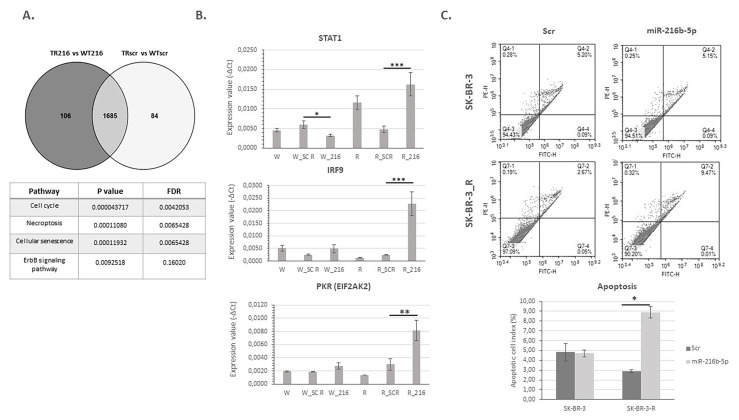
Up-regulation of miR-216-5p induced apoptosis in trastuzumab resistant SK-BR-3 cells. A. 106 genes, obtained from microarray analysis were found to be differentially expressed specifically in trastuzumab resistant cells (TR216). Pathway enrichment analysis was performed with these genes. B. The expressions of *STAT1, IRF9, and PKR (EIF2AK2)* significantly increased in resistant cells after miR-216b mimic transfection (n = 3, ANOVA; *p < 0.05, **p < 0.01, ***p < 0.001). C. A significant increase in the late apoptotic/necroptotic cell index was observed in resistant cells whereas there was no significant change in sensitive cells (t-test; *p < 0.05). The figure indicates the apoptotic profile following annexin V/PI staining; miR-216b-5p mimic increased apoptosis in SB-BR-3-R cells. The histogram indicates the cell index of apoptotic cells.

Microarray data analysis was performed to find out the differentially expressed genes between miR-216b-5p mimic and scrambled control transfected WT cells (W_216b_, W_Scr_) and TR cells (R_216b_, R_Scr_). Intersection of the gene lists were determined with Venny (Oliveros 2007) and 106 genes were differentially expressed specifically in TR cells. According to the anticancer activity results, miR-216b-5p was effective in TR cells rather than WT cells. Therefore, we concentrated on 106 genes for further analysis. Pathway enrichment analyses performed by this gene set revealed cell death related pathways as necroptosis, cell cycle, and cellular senescence (Figure 3A).

Three genes; *STAT1, IRF9*, and *PKR (EIF2AK2)* were significantly enriched in the necrosome formation in the necroptosis pathway (Supplementary S2). Microarray results showed an increase in the *STAT1, IRF9, and PKR (EIF2AK2)* expression in miR-216b-5p mimic transfected resistant cells. The results were validated by qRT-PCR. Expression levels of *STAT1, IRF9, and PKR (EIF2AK2)* significantly increased after miR-216b-5p mimic transfection in TR cells. On the other hand, *STAT1* expression decreased after miRNA transfection in sensitive cells, supporting its anticancer effect in TR cells. The decrease in the expression levels of *IRF9 and PKR (EIF2AK2)* as the cells gain resistance to trastuzumab and the increase after mimic transfection supports miR-216b-5p’s predicted opposing effect to the resistance mechanism (Figure 3B).

Pathway enrichment results together with qRT-PCR validations led us to perform apoptosis analysis with WT and TR cells transfected with scrambled control and mimic. The analysis showed that resistant cells transfected with miR-216b-5p were significantly in the late apoptosis/necrosis stage compared to scr transfected control cells (Figure 3C). miR-216b-5p transfection did not promote cell death in WT cells supporting our hypothesis that miR-216b-5p has an opposing effect in resistance mechanism.

## 4. Discussion

Accumulating investigations have highlighted that miRNAs are involved in the pathogenesis and drug resistance mechanism of breast cancer (Floros et al., 2018; Cataldo et al., 2020; Ma and Zhou 2020; Grimaldi, Salvatore, and Incoronato 2021; Pan et al., 2021; Z. Zhang et al., 2021; Zhou et al., 2021). For instance, PTEN downregulation targeted by miR-21, conferred trastuzumab resistance in the breast cancer cell line, MDA-MB-453. Inhibition of miR-21 could resensitized to trastuzumab by inducing PTEN expression in drug-resistant breast cancer (Gong et al., 2011). FOXO3a accelerated the trastuzumab resistance of HER2-positive breast cancer through targeting and up-regulating IGF2/IGF-1R/IRS1 signaling (Luo et al., 2021). As for miR-126, Fu and Tong found that lower miR-126 facilitated the ability of migration and invasion of trastuzumab-resistant breast cancer cells through targeting miR-126/PIK3R2 axis (Fu and Tong 2020). As mentioned above, miRNAs are considered to have great potential in the treatment of resistant breast cancer. Here, we identified novel findings to illustrate the mechanism of miR-216b-5p in trastuzumab resistance.

The tumor suppressive role of miR-216b has been shown in colon and lung cancer, cervical cancer, and pancreatic cancer; suppressing proliferation, invasion, and migration, being a marker of good prognosis, and preventing the colony formation of the cancer cells, respectively

(S. He et al., 2017; Ferino et al., 2018; Yao, Li, and Wang, 2018; L. Wang et al., 2019). The effect of miR-216b-5p in drug resistance was shown in different cancers (J. He et al., 2019; Pei, Zhao, and Shuang, 2020; P. Wang et al., 2022; Xing et al., 2022). A study reported that a low miR-216b expression negatively affects the response to radiotherapy in pancreatic cancer (Egeli et al., 2016). There are also other studies showing that miR-216b increases the sensitivity to drugs such as to oxaliplatin in colon cancer and to cisplatin in ovarian cancer (Liu et al., 2017; Zou et al., 2017). miR-216b-5p was also shown to have tumor suppressor role of in breast cancer (Menbari et al., 2019) but the regulative role of this miRNA in trastuzumab treatment is not studied. In our previous bioinformatics-based study miR-216b-5p was found to take central role in trastuzumab response by being a hub miRNA in a network of trastuzumab responsive miRNAs in breast cancer cells (Cilek et al., 2017). In the current study, we first identified an anticancer effect of miR-216b-5p on trastuzumab resistant SK-BR-3 cells. Interestingly, overexpression of miR-216b-5p decreased the proliferation, motility, and invasion abilities of resistant cells but not that of sensitive cells. Also, with the increased expression of miR-216b-5p, resistant cells restored sensitivity to trastuzumab.

The drug resistance has been related to cellular processes such as apoptosis, cell cycle or crosstalk between receptor signaling pathways, and the regulative roles of microRNAs has been investigated. Apoptosis, programmed necrosis and autophagy are the three main forms of programmed cell death (Bialik et al., 2010; Chen et al., 2010; McCall, 2010) and are therapeutic targets in anticancer drug resistance. Apoptosis does not have only a single effect on breast cancer resistance. Activation of apoptosis induced by miRNAs increases proliferation inhibition in breast cancer cells. Recent studies reported that increased apoptosis via regulation of miRNAs expression induced cell death and increased the sensitivity of breast cancer cells to drugs (Cittelly et al., 2010; Jiang et al., 2018; W. Zhang et al., 2019; Citron et al., 2020). After performing microarray experiments to enlighten the molecular mechanisms underlying the observed anticancer effects in resistant cells, cell death related pathways such as necroptosis, cellular senescence, and cell cycle were obtained following the pathway analyses performed with the differentially expressed gene sets. The enrichment of the genes *STAT1, IRF9*, and *PKR (EIF2AK2)* in all the pathways and their key roles in necrosome formation in necroptosis pathway led us to concentrate on these three genes and necroptosis pathway. In a study, the importance of *STAT1 and PKR* in IFN-γ dependent necrosome formation was emphasized. The study reported that IFN-γ uses Jak/STAT pathway to induce necrosis and they pointed out that cells show resistance to IFN-γ induced necrosis in the presence of a PKR inhibitor (Thapa et al., 2013). In addition to these findings, the significance of IRF9 was reported in IFN-β induced necroptosis of macrophages (McComb et al., 2014). Considering these previous findings, our demonstration of an upregulation of *STAT1, IRF9, and PKR (EIF2AK2)* in miR-216b-5p transfected resistant cells supports necroptosis related anticancer effect of miR-216b-5p in trastuzumab resistant cells. Concordantly our apoptosis analysis showed that a significant amount of trastuzumab resistant SK-BR-3 cells entered to late apoptosis/necrosis stage upon miR-216b-5p overexpression, which may explain the anticancer effect of miR-216b-5p in trastuzumab resistant cells.

The regulation of specific miRNAs could improve response to treatment and acquired resistance affecting the survival rates of breast cancer patients. Hence, understanding the role of microRNAs, their targets, and the signaling pathways that they are taking part in could extend their usage in clinic. Related to this knowledge it could be concluded that reexpression of miR-216b-5p sensitizes trastuzumab resistance through necroptosis in breast cancer.

## Supplementary Information

Supplementary S1 miR-216b-5p overexpression decreased the invasiveness of trastuzumab resistant SK-BR-3 cells compared to scrambled control transfected cells.

Supplementary S2 KEGG figure showing the necroptosis pathway, the genes obtained from the microarray data were shown in red.

**Supplementary Table 1:** Sequences of primers for GAPDH, STAT1, PKR (EIF2AK2), IRF9, miR-216b-5p, and RNU6.

Gene ID	Sequences (5′to 3′)	RefSeq ID
GAPDH	F: GTTCGACAGTCAGCCGCAT	NM_002046.7
	R: TGAAGGGGTCATTGATGGCA	
STAT1	F: GTGGTACGAACTTCAGCAGC	NM_007315.4
	R: CAGCTGTGACAGGAGGTCAT	
PKR (EIF2AK2)	F: CAGTTTGCCTTCCTGGATTTGT	NM_001135651.3
	R: TACTCCCTGCTTCTGACGGT	
IRF9	F: ACCAGGATGCTGCCTTCTTC	NM_006084.5
	R: CCTGGTGGCAGCAACTGATA	
miR-216b-5p	AAATCTCTGCAGGCAAATGTGA	MIMAT0004959
RNU6-1	AACGCTTCACGAATTTGCGT	-
