# Supplementary figures and images for: miR-216b-5p promotes late apoptosis/necroptosis in trastuzumab-resistant SK-BR-3 cells
Source: Turk J Biol. 2023 May 23;47(3):199–207. doi: 10.55730/1300-0152.2655 (PMC10387915; doi:10.55730/1300-0152.2655)

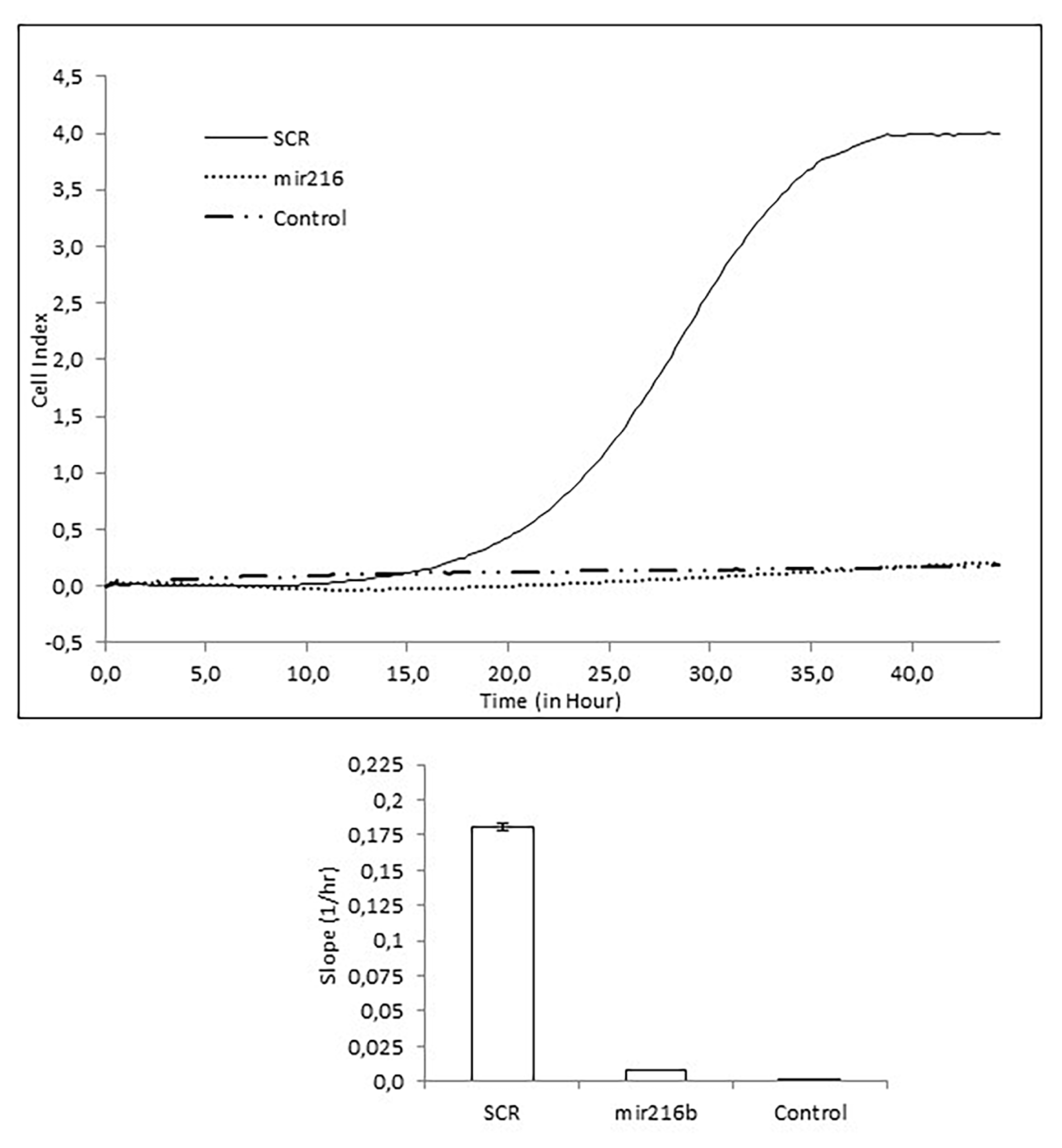

Supplement: Supplementary S1 — miR-216b-5p overexpression decreased the invasiveness of trastuzumab resistant SK-BR-3 cells compared to scrambled control transfected cells. [file turkjbiol-47-3-199s1.tif]

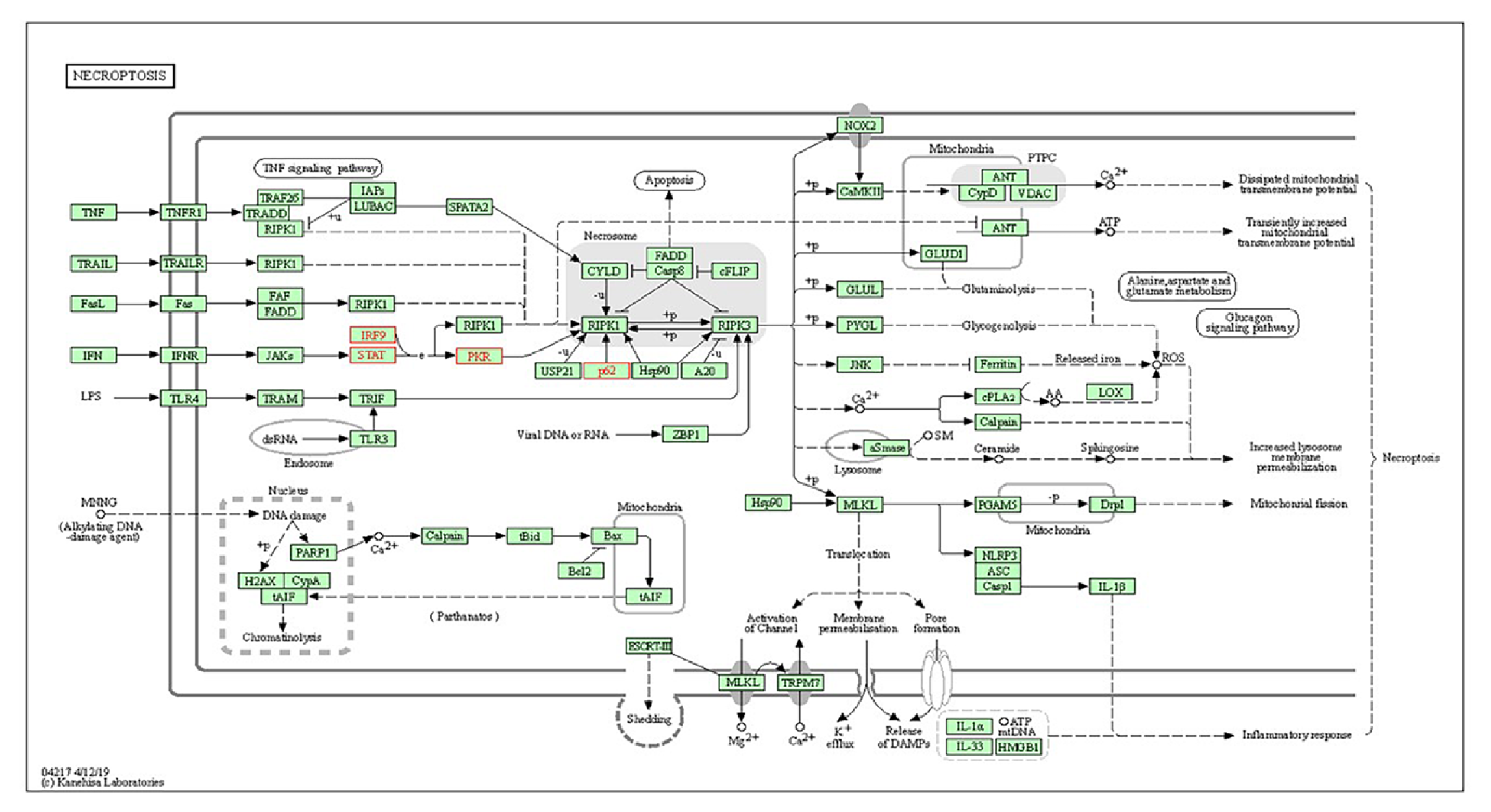

Supplement: Supplementary S2 — KEGG figure showing the necroptosis pathway, the genes obtained from the microarray data were shown in red. [file turkjbiol-47-3-199s2.tif]
